# *Candidatus* Liberibacter asiaticus encodes a functional BolA transcriptional regulator related to motility, biofilm development, and stress response

**DOI:** 10.3389/fmicb.2026.1717228

**Published:** 2026-01-29

**Authors:** Xuanlin Zhan, Guoyi Huang, Jun Su, Jingtian Zhang, Qiting Huang, Xiaoling Deng, Meirong Xu

**Affiliations:** 1 Guangdong Province Key Laboratory of Microbial Signals and Disease Control, Citrus Huanglongbing Research Laboratory, South China Agricultural University, Guangzhou, China; 2 National Center for International Collaboration Research on Precision Agricultural Aviation Pesticide Spraying Technology, Guangdong Engineering Technology Research Center of Smart Agriculture, College of Artificial Intelligence, South China Agricultural University, Guangzhou, China

**Keywords:** citrus greening, gene function, heterologous expression, huanglongbing, inhibitor screening, virulence

## Abstract

“*Candidatus* Liberibacter asiaticus” (CLas) is an uncultivable *α*-proteobacterium causing the most destructive and currently incurable citrus disease, Huanglongbing (HLB). The transcription factors (TFs) of CLas are involved in various biological processes. However, the functions of most TFs remain unverified. BolA is reported to be an important transcriptional regulator related to bacterial growth and virulence. Here, the role of BolA in CLas was investigated using gene deletion and complementation assays in the heterologous host *Sinorhizobium meliloti* (*Sme*). The results showed that *BolA*_CLas_ and *BolA_Sme_* are similar in sequence and transcriptional regulation. BolA positively regulates biofilm formation—evidenced by the significant downregulation of a key gene (*cyaA*) in the mutant (Δ*BolA_Sme_*), without affecting bacterial growth. The upregulation of 16 differentially expressed genes (DEGs) related to flagellar assembly indicated that BolA negatively regulates CLas motility. *BolA* deletion also led to the downregulation of ABC transporters (15 DEGs) and lipid metabolism genes (13 DEGs), correlating with reduced stress tolerance. Furthermore, *BolA*_CLas_ is involved in modulating heme metabolism, as well as protein export, folding, sorting, and degradation. Finally, *in vivo* screening identified two compounds as BolA inhibitors, which significantly reduced CLas titer in infected periwinkle leaves. Taken together, this study constitutes a relevant step toward the understanding of CLas virulence by demonstrating that BolA is a key TF involved in biofilm formation, stress response, motility, and bacterial physiology, thereby presenting a potential target for disease control.

## 1 Introduction

Citrus Huanglongbing (HLB) is the most destructive disease, currently devastating the citrus industry worldwide. HLB is widely distributed throughout tropical and subtropical regions of Asia, America, and Africa (Wang and Trivedi, 2013). It causes significant yield losses in affected areas (Nehela and Killiny, 2020). Under the ravages of HLB, no effective chemicals except antibiotics such as streptomycin and oxytetracycline have been used to eliminate HLB symptoms in affected trees (Barnett et al., 2019). However, the duration of the therapeutic effect is limited, and side effects on human beings are still unknown without rigorous testing. Consequently, multipronged management strategies, including the eradication of diseased trees, controlling the insect vectors, and a variety of agricultural control measures, are currently applied to control HLB.

The presumed causal agent of HLB, “*Candidatus* Liberibacter spp.,” within the family Rhizobiaceae of the order Rhizobiales (Garnier et al., 2000). “*Candidatus* Liberibacter asiaticus” (CLas) is the most prevalent form of HLB pathogen worldwide. The relatively reduced genome of CLas is approximately 1.26 Mb in size, encoding about 1,100 genes. Genome annotation results indicate that CLas can metabolize fructose, glucose, and xylose, but not mannose, galactose, rhamnose, or cellulose (Duan et al., 2009). CLas also lacks many genes involved in biosynthesis pathways, indicating that it should be a parasitic bacterium that acquires some micronutrients from the phloem of its host plants or vectors. Furthermore, CLas lacks genes encoding virulence factors for type III and type IV secretion systems but possesses genes associated with the type I secretion system and the Sec secretion system (Duan et al., 2009; Wang and Trivedi, 2013).

*Sinorhizobium meliloti* (*Sme*) is a rhizobia-related bacterium with a genome size of approximately 6.7 Mb. It belongs to the family Rhizobiaceae and is experimentally used as a heterologous host to study the protein functions of CLas. Given the uncultivable nature of CLas, the validity of using *Sme* as a surrogate host has been well established, including for transcription factors (TFs) (Barnett et al., 2019; Wang et al., 2025). The *Sme* genome comprises a 3.65 Mb chromosome, a 1.35 Mb megaplasmid pSymA, and a 1.68 Mb megaplasmid pSymB (Galibert et al., 2001). This cultivable bacterium contains genes involved in various biosynthesis pathways, including nucleic acid and protein metabolism, motility, chemotaxis, plant interaction (putative virulence genes), and stress response (Capela et al., 2001), which shares similarity with those of CLas.

Recently, the gene function of fastidious CLas has been widely studied using genetically tractable, heterologous hosts *Sme* and *L*. *crescens*. Vahling-Armstrong et al. (2012) employed a complementation assay in *Sme* to study the role of CLas znu ABC proteins and found that only one of the two Znu ABC in CLas could restore the zinc absorption activity of the *Znu ABC* mutant. The authors suggested that these different zinc absorption regulation modes in CLas are important for the pathogen’s growth and virulence, potentially contributing to the zinc deficiency-like symptoms in HLB-affected plants. Zou et al. (2012) studied the function of a flagellin and hook-associated protein (Fla) of CLas with *Sme*, and concluded that Fla. acts as a PAMP that might play an important role in triggering host plant resistance to the CLas. Mutation of the *LdtR* gene in *L*. *crescens* resulted in shortened cell morphology and reduced tolerance to osmotic stress. Furthermore, LdtR was shown to regulate CLas gene transcription and has been implicated in processes such as cell wall biogenesis, cell motility, energy production, and zinc uptake (Pagliai et al., 2018).

Some bacterial TFs are virulence-associated (Occhialini et al., 2005). Targeting these TFs with inhibitors represents a promising, target-based strategy for antimicrobial discovery, offering a more direct path than phenotypic screening. Wang et al. (2025) recently characterized the TF Rem in CLas, demonstrating its role in regulating flagellar assembly and motility through heterologous complementation in *Sme*, thereby providing new insights into the regulatory mechanisms of CLas during infection in the host. Barnett et al. (2019) designed a synthetic system for high-throughput screening inhibitors of CLas transcription regulators *in vitro* and *in vivo*. The study identified dozens of potential inhibitors, such as ChemDiv C549-0604, which continuously and strongly inhibited a green fluorescent protein reporter under the control of the CLas activator VisNR (IC50 = 0.7 μM), and decreased the motility of *Sme*Δ*VisNR* CLas p*VisNR* (a complementation strain) by 28% without affecting cell viability.

BolA is an important transcriptional regulator found in most Gram-negative bacteria (Dressaire et al., 2015) that is involved in modulating flagella biosynthesis, biofilm development, and stress adaptation (da Silva et al., 2023). It is also involved in Fe-S cluster biogenesis, storage, trafficking, and signaling that control iron metabolism (Talib and Outten, 2021). Furthermore, BolA has been suggested to play a potential role in antibiotic response (Aldea et al., 1989). In *Salmonella enterica* Serovar Typhimurium, BolA is a master virulence factor that helps the strain overcome the host immune system (Mil-Homens et al., 2018). Similarly, it influences the virulence and stress response of *Klebsiella pneumoniae* (Zhang et al., 2022). Recently, Zheng et al. (2023) found differences in pathogenicity between two CLas strains, namely PYN from high-altitude areas and PGD from low-altitude areas. BolA is one of the four transcriptional regulators that were found to be induced in the relatively mild strain PYN (fold change = 5.2). Therefore, it is speculated that BolA may regulate the stress response and influence the virulence of CLas.

In this study, we elucidated the function of CLas BolA using *Sme* as a heterologous host. Subsequent transcriptome analysis was performed to elucidate its molecular regulatory mechanisms. Molecular inhibitors were screened by homology modeling and virtual screening. The functional characterization of BolA, combined with inhibitor screening, represents a significant step toward understanding the molecular mechanisms of this pathogen and provides future perspectives for HLB treatment.

## 2 Materials and methods

### 2.1 DNA or RNA extraction, and cDNA synthesis

Genomic DNA and RNA samples of the bacteria (*Sme*) were extracted using a bacterial genomic DNA extraction kit and an RNA kit (both from Omega Bio-Tek, Norcross, United States), respectively, following the manufacturer’s instructions. The purity and concentration of DNA or RNA were assessed using a NanoDrop™ One (Thermo Scientific, Shanghai, China). Qualified RNA samples were then treated with a TransScript^®^ One-Step gDNA Removal kit and reverse transcribed into cDNA using cDNA Synthesis SuperMix (TransGen Biotech, Beijing, China).

For gene expression analysis of CLas within plant hosts, total RNA was extracted from the midribs of CLas-infected citrus leaves using the Plant RNA kit (Omega Bio-Tek). Reverse transcription was performed with a standardized input of 1,000 ng of total RNA, using the same TransScript® SuperMix. This kit employs a mixture of random hexamers and anchored oligo (dT)₁₈ primers, ensuring efficient cDNA synthesis from both bacterial transcripts (via random priming) and plant host mRNAs (via oligo-dT priming). No poly-A tail addition step was involved, consistent with the prokaryotic nature of CLas.

The integrity and functionality of all synthesized cDNA were confirmed by the successful and consistent amplification of target reference genes (e.g., gyrA for CLas) in subsequent qPCR analyses.

### 2.2 Construction of *BolA_Sme_* mutant in *Sme*

All the primers used in this study are listed in Supplementary Table S1. PCR amplification was performed using PrimeSTAR^®^ Max DNA Polymerase (TaKaRa Biotek, Beijing, China) according to the manufacturer’s instructions. Gene deletion was performed via homologous recombination using the suicide vector pK18mobsacB. Briefly, the upstream and downstream homology arms of *BolA_Sme_* were amplified with primer pairs BolA-UF/R and BolA-DF/R, respectively, using the genomic DNA of *Sme* Rm1021 as the template. The purified fragments were fused by overlap extension PCR to generate a 1,019-bp fusion fragment. This fusion product and the pK18mobsacB plasmid were digested with *Bam*HI and *Xba*I (Takara, China) in a 20 μL reaction mixture containing 1 μL of each restriction enzyme, 2 μL of 10 × QuickCut Buffer, and 16 μL of DNA template. The digestion was carried out at 37 °C for 25 min. The digested fusion fragment was then ligated into the linearized pK18mobsacB vector using T4 DNA Ligase (Takara Biotek, Beijing, China). The ligation product was transformed into competent *E. coli* DH5α cells via heat shock. The resulting recombinant plasmid was verified by PCR and Sanger sequencing.

The mutant (Δ*BolA_Sme_*) was constructed via tri-parental conjugation. Briefly, donor *E. coli* (carrying pK18mobsacB/*BolA_Sme_*), helper *E. coli* (carrying pRK2013), and the recipient *Sme* Rm1021 were mixed at a ratio of 1:1:2 (v/v). The mixed cell cultures were pelleted by centrifugation, washed three times with sterile ddH2O to remove antibiotics, resuspended, and spotted onto antibiotic-free TY plates. After a 2-h upright incubation at 28 °C to allow mating initiation, the plates were inverted and incubated for 3 days. Conjugants were selected on TY plates containing gentamicin (Gm, 50 μg/mL) and nalidixic acid (Na, 100 μg/mL). Putative single-crossover integrants were inoculated into TY liquid medium with Na (100 μg/mL) and cultured for 24 h. The cultures were then diluted and plated on TY plates containing 10% (w/v) sucrose to select for double-crossover events. Sucrose-resistant colonies were initially screened by colony PCR using primers of pK18-cx-F/R. The *BolA_Sme_* gene deletion was ultimately confirmed by Sanger sequencing, yielding the verified mutant strain Δ*BolA_Sme_*.

### 2.3 Construction and identification of the *BolA_CLas_* complementation strain in *Sme*

For heterologous gene expression, the full-length coding sequence of the *BolA*_CLas_ gene was amplified from the genomic DNA of CLas-infected periwinkle leaves using primers CLas-*BolA*-F/R (Zheng et al., 2023). The purified PCR product was cloned into the *BamH*I/*Xba*I-digested shuttle vector pBBR1MCS-4 plasmid using the ClonExpress II One Step Cloning kit (Vazyme Biotech., Beijing, China), yielding the recombinant plasmid pBBR1MCS-4/*BolA*_CLas_. In this construct, *BolA*_CLas_ expression is driven by the native promoter of the pBBR1MCS-4 vector. The plasmid construct was verified by PCR using MCS-F/R primers and by Sanger sequencing. The complementation strain *BolA*_CLas_/Δ*BolA_Sme_* was generated by introducing pBBR1MCS-4/*BolA*_CLas_ into the Δ*BolA_Sme_* mutant via tri-parental conjugation, as described in Section 2.2. Transconjugants were selected on TY plates containing 50 μg/mL ampicillin (Amp) and 100 μg/mL Na for 3 days. Successful construction of the complementation strain was confirmed by PCR amplification of the *BolA*_CLas_ insert using primers MCS-F/R, followed by Sanger sequencing.

### 2.4 Phenotype determination

The wild-type Rm1021, the Δ*BolA_Sme_* mutant, and the *BolA_CLas_*/Δ*BolA_Sme_* complementation strain were pre-cultured in TY medium with appropriate antibiotics to the exponential phase. For each strain, single colonies were individually picked and inoculated into 10 mL of TY liquid medium containing the respective antibiotics, and incubated at 28 °C with shaking at 200 rpm for 36 h. The cultures were then diluted to an initial OD_600_ of 0.1. A 1 μL aliquot of each diluted culture was used to inoculate 100 μL of fresh TY medium in each well of the PVC-96 well plate. The plate was incubated at 28 °C with shaking at 200 rpm. The OD_600_ was measured every 2 h for 80 h using a BioMate 3S ultraviolet–visible spectrophotometer (Thermo Fisher Scientific, Inc., Waltham, MA, United States). Fresh TY medium was served as the blank control. The experiment was performed with three independent biological replicates.

For the biofilm formation assay, overnight cultures of the three strains were adjusted to an OD_600_ of approximately 0.1. A 5 μL aliquot of each adjusted culture was inoculated into test tubes containing 4 mL of fresh TY medium and incubated statically at 28 °C for 7 days. After incubation, the biofilm was stained with 1% crystal violet solution for 20 min. Unbound dye was removed by rinsing twice with deionized water. The stained biofilm was dissolved in 4 mL of 95% ethanol, and the absorbance of the solution was measured at 490 nm (OD_490_) using a spectrophotometer (Multiskan GO 1510, ThermoFisher, Finland). The experiment included three biological replicates.

For the motility assay, bacterial cultures were adjusted to an OD_600_ of approximately 0.5. A 3 μL aliquot of each culture was spot-inoculated onto the center of TY agar plates containing 0.3% agar. The plates were incubated at 28 °C for 48 h. Motility was quantified by measuring the diameter (in millimeters) of the circular migration zone formed by the bacteria. The experiment was repeated three times independently.

### 2.5 Transcriptome sequencing and analysis

Transcriptome sequencing was performed by Novo Biomedical Technology Co., Ltd. Three independent biological replicates of three samples, the wild-type Rm1021, the Δ*BolA_Sme_* mutant, and the *BolA*_CLas_/Δ*BolA_Sme_* complementation strain, were prepared. Total RNA quality was assessed using a NanoDrop™ instrument, and RNA integrity was verified using an Agilent 2,100 Bioanalyzer. Sequencing libraries were constructed using a Total RNA Library kit (Omega Bio-Tek, Norcross, USA) according to the manufacturer’s instructions and sequenced on an Illumina platform to generate 150-bp paired-end reads.

Raw sequencing data were processed to obtain clean data. Briefly, adapter sequences and low-quality bases were trimmed using Cutadapt. Reads with an average quality score below Q20 or a length of less than 50 bp after trimming were discarded. The clean reads were then aligned to the complete reference genome of *Sme* Rm1021 (strain ATCC 51124/1021; NCBI Taxonomy ID: 266834; Assembly: ASM696v1) using Bowtie2 (v2.3.5.1). Read counts for each gene were generated using HTSeq (v0.13.5). Differential gene expression analysis was performed using the DESeq2 (v0.13.5) package in R. Genes with an absolute |log_2_FoldChange| > 0.6 adjusted *p*-value (FDR) < 0.05 were considered differentially expressed. For functional annotation, KEGG pathway enrichment analysis was carried out using the R-package “clusterProfiler” (v4.0.5), with *Sme* Rm1021 as the background and the KEGG database (Release 104.1). Enriched pathways were identified with a significance threshold of p-value < 0.05. Protein–protein interaction (PPI) networks were predicted using the STRING database (v11.5) for *Sme* Rm1021, with a minimum required interaction score set at 0.7 (high confidence).

### 2.6 Homology modeling, virtual screening, and molecular docking

The three-dimensional structure of the CLas BolA protein was predicted by homology modeling using the SWISS-MODEL online server (Waterhouse et al., 2018). The protein sequence of CLas BolA was submitted to the server, which automatically selected the AlphaFold DB model of A0A094Z2Y7_9HYPH (a BolA family protein from *Candidatus* Liberibacter solanacearum) as the template based on high sequence identity (73.74%) and model quality assessment. The quality of the generated model was evaluated using the built-in GMQE score (0.88).

Prior to docking, the BolA protein model was prepared using the ‘Protein Preparation’ module in Discovery Studio 2019 (BIOVIA). This process involved adding hydrogen atoms, removing water molecules, and optimizing side-chain conformations and hydrogen bonding networks. A library of small molecules from HTS Biochemie Innovationen was prepared using the ‘Prepare Ligands’ module, which standardized the structures, enumerated possible tautomers, and generated 3D conformations.

Molecular docking was performed to predict the binding modes and affinities of the small molecules to the BolA model. Docking simulations were carried out using the ‘Dock Ligands (LibDock)’ protocol within Discovery Studio. The binding site was defined to encompass the predicted active pocket of BolA. All other parameters were kept at the default settings of the software. The docking poses were ranked based on their LibDock scores, and the top-ranking compounds were selected for subsequent experimental validation.

### 2.7 Determination of minimum inhibitory concentration against *Sinorhizobium meliloti*

Given the uncultivable nature of CLas, the MIC of the selected compounds was first determined against the culturable surrogate bacterium, *Sme*, using the broth microdilution method, as previously described (Xu et al., 2022), with minor modifications. Briefly, each compound was first dissolved in dimethyl sulfoxide (DMSO) to prepare a 1 mg/mL stock solution. This stock was then subjected to two-fold serial dilutions in a 96-well microtiter plate containing fresh medium.

*Sinorhizobium meliloti* cells from an overnight culture were diluted to a density of approximately 1 × 10^5^ CFU/mL in the same medium, and an equal volume of this bacterial suspension was added to each well containing the serially diluted compounds. The final concentration of DMSO in all wells, including the growth control (bacteria with medium and DMSO only), did not exceed 1% (v/v) and was confirmed to have no effect on bacterial growth. A sterility control (medium only) was also included. The plates were incubated at 30 °C for 48 h.

After incubation, bacterial growth was assessed by measuring the optical density at 600 nm (OD₆₀₀) using a microplate reader. The MIC was defined as the lowest compound concentration at which no visible growth was observed, and the OD₆₀₀ reading was comparable to that of the sterility control.

### 2.8 Assessment of inhibitory effects on CLas in grafted periwinkle plants

To evaluate the *in planta* inhibitory efficacy of the candidate compounds against the uncultivable CLas, a grafting assay was used. In addition, 45-day-old periwinkle seedlings were used as rootstocks. Grafting material (leaves) were collected from CLas-infected detached branches (pre-treatment Ct value = 21.67 ± 0.31) that had been hydroponically cultured for 20 days in solutions containing either compound B1 (N-[(2-chlorophenyl)methyl]-5-(1-【[cyclohexyl(methyl) carbamoyl] methyl】-2,4-dioxo-1,2,3,4- tetrahydroquinazolin −3-yl) pentanamide) or compound B2 (2-[(【[(furan-2-yl) methyl] carbamoyl】methyl) sulfanyl]-4-oxo-N,3-bis[(oxolan-2-yl) methyl]-3,4-dihydroquinazoline-7-carboxamide). Branches from the same plant maintained in sterilized water served as the control. After 20 days, leaves from each of the three branch types were individually grafted onto the apex of periwinkle seedlings, with six biological replicates per treatment. The grafted plants were subsequently maintained in a screenhouse for an additional 45 days. Successful CLas infection was confirmed by quantifying bacterial titers using qPCR. This molecular approach is the established standard for definitively assessing colonization in this pathosystem, as visible disease symptoms (e.g., leaf chlorosis) often develop slowly and inconsistently within experimental timeframes.

For DNA extraction, midribs from periwinkle leaves were cut into fine pieces and mechanically ground in the extraction solution provided with a commercial plant DNA extraction kit (E. Z. N. A.® HP Plant DNA kit, D2485-04). DNA was extracted following the manufacturer’s protocol. qPCR was performed to quantify CLas titers using the extracted DNA as template, as described by Bao et al. (2020).

## 3 Results

### 3.1 *BolA*_CLas_ and *BolA_Sme_* exhibit sequence and transcriptional similarities

**Figure 1:**
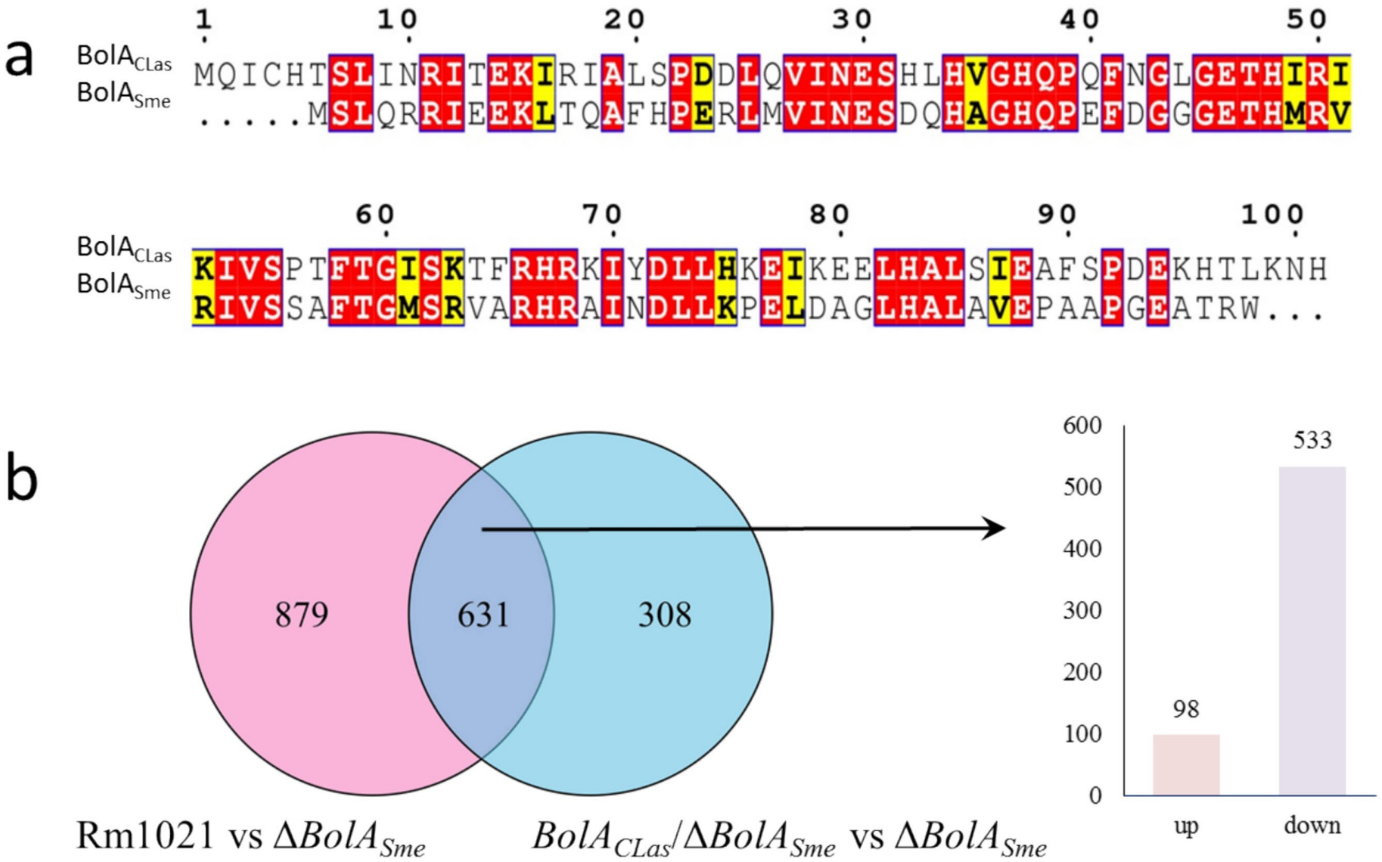
Sequence alignment and transcriptomic analysis of BolA. **(a)** Amino acid sequence alignment of BolA_CLas_ and BolA*_Sme_*, note: Identical residues are highlighted in red; residues with similar biochemical properties are highlighted in yellow. **(b)** Venn diagrams showing numbers of differentially expressed genes (DEGs). Rm1021 *vs* Δ*BolA_Sme_*: compares the wild-type strain Rm1021 to the Δ*BolA_Sme_* mutant. *BolA_CLas_*/Δ*BolA_Sme_ vs* Δ*BolA_Sme_*: compares the complemented strain *BolA*_CLas_/Δ*BolA_Sme_* to the Δ*BolA_Sme_* mutant.

The *BolA* in the A4 strain of CLas is 306 bp in length, encoding 101 amino acids. Comparatively, the *BolA* in the Rm1021 strain of *Sme* (*BolA_Sme_*) is 282 bp and encodes a 93 amino acid protein. Sequence alignment revealed 73.85% amino acid identity between the two proteins (Figure 1a). A4 encoded BolA (C_522_H_838_N_152_O_149_S_2_) has a molecular weight of approximately 11.69 kDa. The computed isoelectric point value of *BolA*_CLas_ is 8.01, and the instability index value is 44.04, indicating that it is an unstable protein. The BolA protein comprises 19 different amino acids, including 12 negatively charged residues and 13 positively charged residues. The BolA_CLas_ is predicted as a hydrophilic protein, with the average hydrophilicity of −0.386.

To construct the Sme BolA mutant, upstream and downstream homology arms (509 and 510 bp, respectively) of *BolA_Sme_* were amplified. These fragments were fused by overlap extension PCR using primers BolA-UF and BolA-DR to generate a 1,019 bp fusion product *BolA_Sme_*. This fragment was cloned into pK18mobsacB (Schafer et al., 1994) to construct a suicide plasmid pK18mobsacB/*BolA_Sme_*, which was verified by PCR using primers of pK18-cx-F and pK18cx-R and by sequencing. The suicide plasmid was then transformed into wild-type *Sme* Rm1021 to generate Δ*BolA_Sme_*. PCR with primers of BolA-UF and BolA-DR generated a 1,019 bp product for Δ*BolA_Sme_*, which was shorter than that of Rm1021 (1,299 bp). The sequencing results of the PCR product further confirmed the successful deletion of *BolA* in *Sme* (Supplementary Figure S1a).

For complementation, a 648 bp fragment containing the *BolA*_CLas_ coding sequence was amplified from the CLas A4 with primers of CLas-BolA-F/R. This fragment was cloned into pBBR1MCS-4, yielding pBBR1MCS-4/*BolA*_CLas_. This recombination plasmid was transferred to Δ*BolA_Sme_* to generate the complementation strain *BolA*_CLas_/Δ*bolA_Sme_*. Both the PCR using primers of MCS4-F and MCS4-R and the sequencing of the PCR products verified the complemented strain *BolA*_CLas_/Δ*BolA_Sme_* was successfully created (Supplementary Figure S1b).

To elucidate the molecular function of *BolA*, RNA-seq analysis was performed. Transcriptional levels of Δ*BolA_Sme_* and *BolA_CLas_*/Δ*BolA_Sme_* were compared to those of the wild-type strain Rm1021. We identified 1,510 DEGs when comparing Rm1021 **to** Δ*BolA_Sme_* (Rm1021 *vs.* Δ*BolA_Sme_*) and 939 DEGs in *BolA_CLas_*/Δ*BolA_Sme_ vs.* Δ*BolA_Sme_* (Figure 1b). Totally 631 DEGs were common in the two comparisons. Of the 631 common DEGs, 98 DEGs were upregulated in both groups, other 533 DEGs were downregulated in both groups. In addition, 508 common DEGs had similar (within two-fold difference) fold change (FC). These results indicate that *BolA*_CLas_ has a similar regulatory mechanism to *BolA_Sme_*.

KEGG pathway enrichment analysis was performed on the 631 co-DEGs. For the 98 co-upregulated DEGs, pathways for “ABC transporters” (enrichment factor = 5.44, adjusted *p*-value = 2.58 × 10^−6^) and “Membrane transport” (enrichment factor = 5.14, adjusted *p*-value = 2.64 × 10^−6^) were the most significantly enriched. These enriched pathways, which include key genes for substrate-binding and permease components, suggest a potential enhancement in the uptake of nutrients, ions, or other small molecules. Among the 533 co-downregulated DEGs, “Protein export” (enrichment factor = 11.70, adjusted *p*-value = 3.56 × 10^−6^) and “Flagellar assembly” (enrichment factor = 5.06, adjusted *p*-value = 1.81 × 10^−3^) were prominently enriched. The concurrent downregulation of flagellar assembly and motility-related pathways (e.g., “Cell motility”) strongly indicates a suppression of bacterial motility and chemotaxis functions.

### 3.2 *BolA*_CLas_ and *BolA_Sme_* positively regulate the biofilm formation without affecting bacterial growth

**Figure 2:**
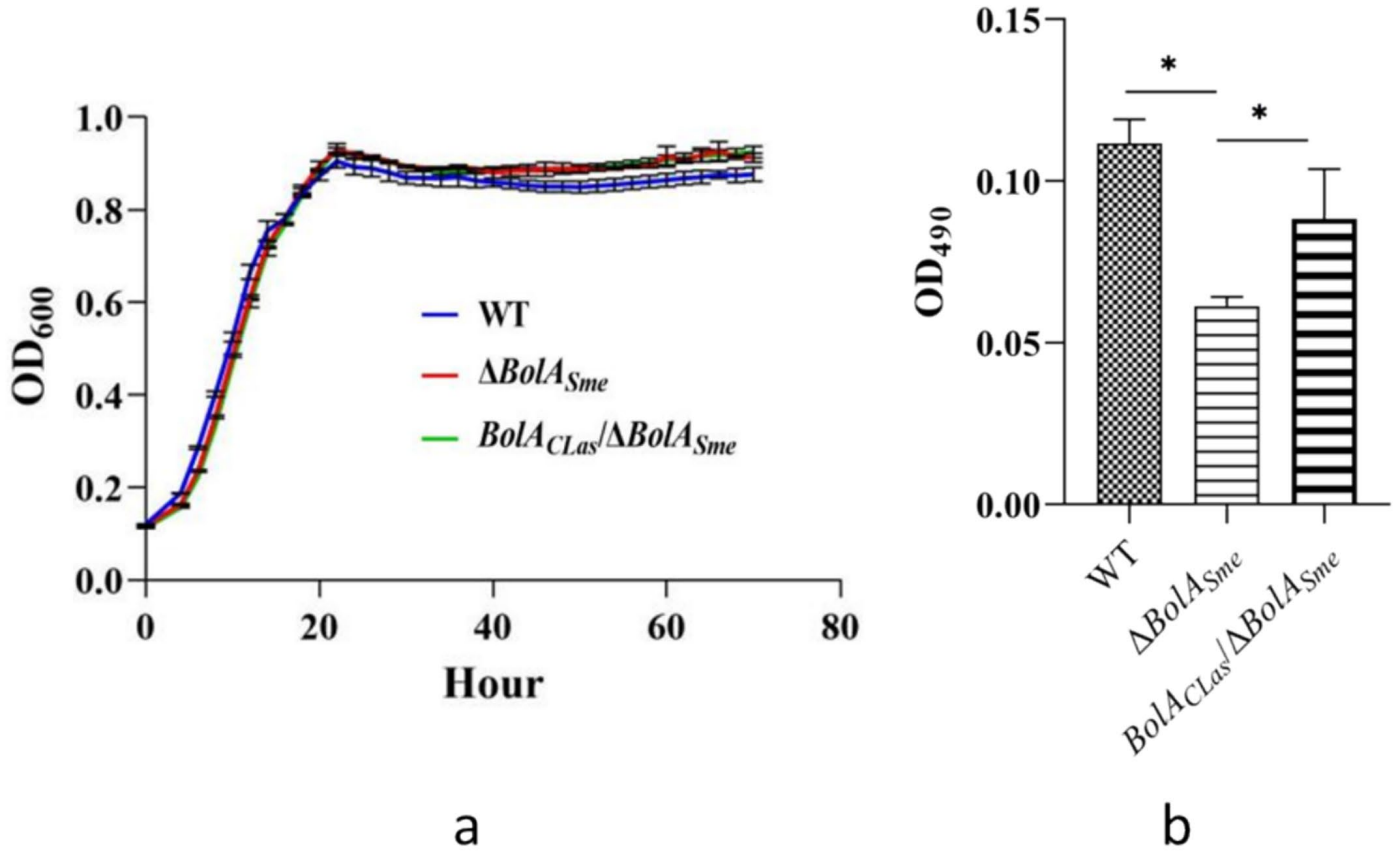
Growth and biofilm formation of *BolA_Sme_* mutant strain (Δ*BolA_Sme_*) and the *BolA_CLas_* complemented strain (*BolA_CLas_*/Δ*BolA_Sme_*). **(a)** The growth curve of Rm1021, Δ*BolA_Sme,_* and *BolA_CLas_*/Δ*BolA_Sme_* in TY medium. **(b)** The role of BolA in the production of biofilm formation. “*” indicates a significant difference between the two sets of data at a level of *p* < 0.05.

Equal amounts (OD_600_ ≈ 0.1) of the *Sme* wild type strain Rm1021, Δ*BolA_Sme_*, and *BolA_CLas_*/Δ*BolA_Sme_* were transferred to fresh TY liquid medium at a 1:100 dilution. All three strains exhibited similar growth kinetics, entering the exponential phase at approximately 20 h post-inoculation (hpi) and maintaining a stationary phase from 20 to 80 hpi. No obvious decline phase was observed within the 80-h monitoring period. Compared to the growth curve of the wild-type strain, the growth curves of the Δ*BolA_Sme_* strain and the complement strain *BolA_CLas_*/Δ*BolA_Sme_* showed no significant difference in growth pattern at all tested time points (Figure 2a). The results showed that neither the deletion of *BolA_Sme_* nor its replacement with *BolA*_CLas_ affects the growth of *Sme*.

Biofilm formation was assessed for the three strains. The Δ*BolA_Sme_* strain produced significantly less biofilm mass than the wild-type strain (*p* < 0.05; Figure 2b). The biofilm formation was partially restored in the *BolA*_CLas_/Δ*BolA_Sme_* complementation strain, being significantly thicker than in the deletion mutant (*p* < 0.05), albeit not fully to the level of the wild-type. These results demonstrate that BolA is essential for normal biofilm formation in both *Sme* and CLas.

To explore the potential mechanism of *BolA* in biofilm formation, we examined the transcriptome data and found that the expression of *cyaA* (SM_RS02400), a key enzyme (adenylate cyclase) involved in the c-di-GMP signaling pathway, was significantly downregulated in the Δ*BolA_Sme_* mutant. Given the established role of c-di-GMP in promoting biofilm formation, the observed reduction in *cyaA* expression provides a plausible transcriptional clue that links BolA to the regulation of this critical signaling pathway. Our data suggest that the biofilm defect in the *BolA* mutant could be associated, at least in part, with alterations in the c-di-GMP pathway, warranting further investigation into the precise mechanism.

### 3.3 *BolA*_CLas_ and *BolA_Sme_* negatively regulated bacterial motility

**Figure 3:**
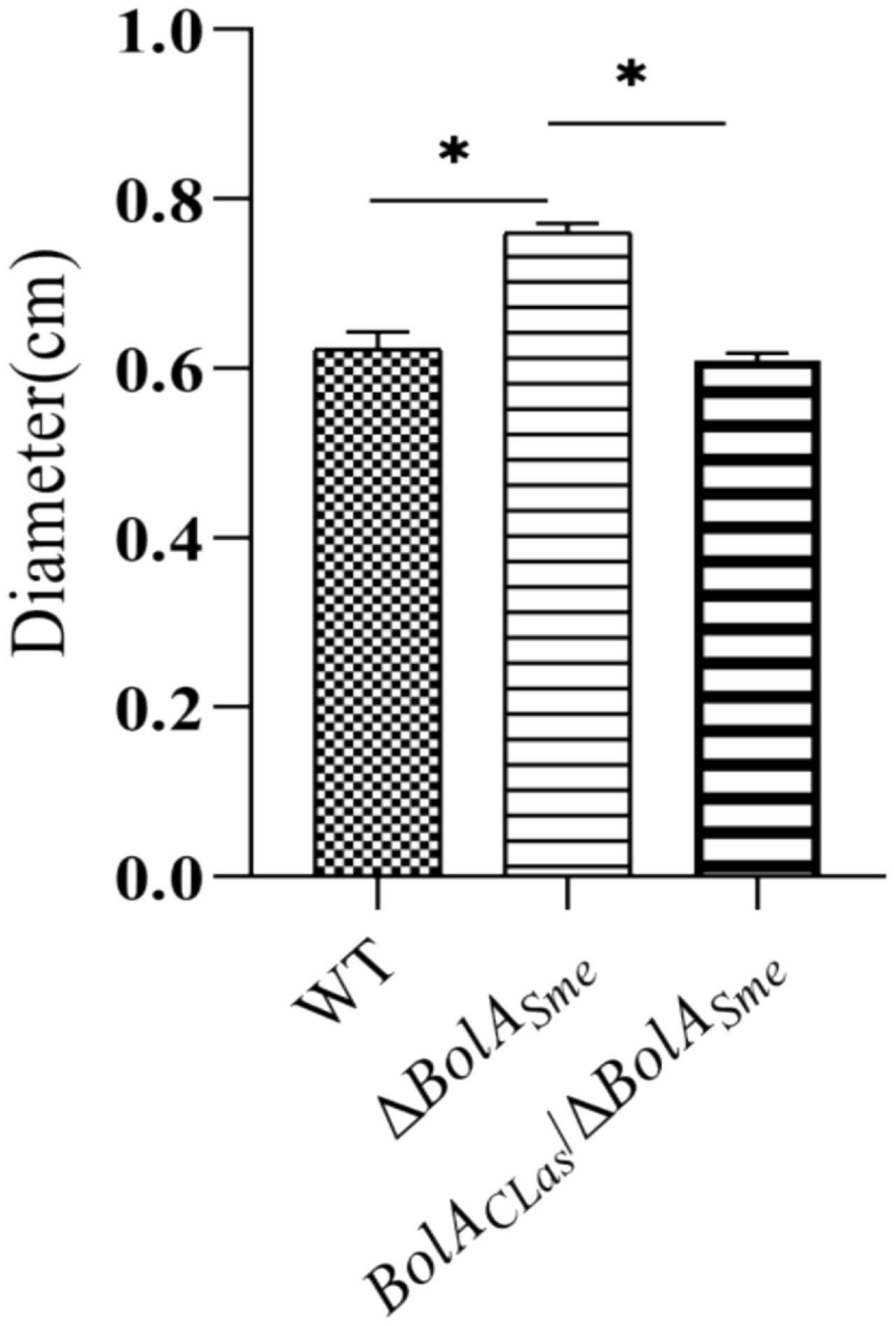
Swimming motility test of the *BolA_Sme_* mutant strain (Δ*BolA_Sme_*) and the *BolA_CLas_* complemented strain (*BolA_CLas_*/Δ*BolA_Sme_*). Note: Graphs are representative of data from three independent experiments; “*” indicates a statistically significant difference (p* < 0.05) between the indicated groups.

Motility test on TY medium with 0.3% agar showed that the Δ*BolA_Sme_* strain had a higher level of circular movement than the wild type strain and the complementation strain (*p* < 0.05). The complemented strain exhibited similar motility to the wild-type strain Rm1021 (Figure 3). The results showed that CLas BolA negatively regulated bacterial motility.

Consistent with this phenotype, DEGs involved in flagellar assembly were all downregulated in both groups (*BolA*_CLas_/Δ*BolA_Sme_ vs.* Δ*BolA_Sme_* and Rm1021 *vs.* Δ*BolA_Sme_*), confirming the negative regulatory role of BolA. Eight such DEGs were identified, including one encoding flagellar basal body P-ring protein, two genes encoding flagellar basal body rod protein (*FlgG* and *FlgC*), one flagellar hook-basal body protein gene (*FliE*), two flagellar motor switch protein genes (*FliN*), and genes *fliM* and *flgI* (Table 1). These flagellar-related DEGs, which were upregulated in the Δ*BolA_Sme_* mutant, are predicted to contribute to bacterial motility processes.

**Table 1:** Differentially expressed genes in the flagellar assembly pathway were suppressed by *BolA_CLas_*.

Gene ID	FC1	FC2	Description
SM_RS03365	0.4	0.36	Flagellar hook-basal body protein FliE
SM_RS03320	0.45	0.32	Flagellar motor switch protein FliN
SM_RS03325	0.5	0.47	Flagellar motor switch transmembrane protein
SM_RS27945	0.25	0.42	Hypothetical protein SMa1195
SM_RS03370	0.44	0.38	Flagellar basal body rod protein FlgG
SM_RS00310	0.56	0.55	Hypothetical protein SMc02580
SM_RS03380	0.53	0.4	Flagellar basal body P-ring protein
SM_RS03360	0.57	0.26	Flagellar basal body rod protein FlgC

FC1, Fold changes of BolA_CLas_/ΔBolA_Sme_ vs. ΔBolA_Sme_; FC2, Fold changes of Rm1021 vs. ΔBolA_Sme_.

### 3.4 BolA enhances stress tolerance by regulating ABC transporters and lipid metabolism

All 15 DEGs associated with ABC transport and membrane transport were induced by BolA (Table 2), including genes involved in the uptake and transport of amino acids, sugars, ions, and lipids. Specifically, BolA appears to regulate ABC transporters for lipids, iron, and nutrients such as amino acids and sugars. A total of 13 DEGs related to lipid metabolism, including those encoding four *gntR*, *VirB5*, *trxB*, *hsrA,* and two *afsQ2* genes, were all downregulated in Δ*BolA_Sme_* (Table 2). These findings suggest that both *BolA_Sme_* and *BolA*_CLas_ positively contribute to lipid metabolism.

**Table 2:** ABC transporter and lipid metabolism-related genes upregulated by BolA.

Pathway	Gene ID	FC1	FC2	Description
ABC transporters	SM_RS16400	1.85	2.16	ABC transporter integral membrane protein
	SM_RS30180	1.99	2.21	ABC transporter permease
	SM_RS21950	1.72	1.92	Iron uptake ABC transporter permease
	SM_RS20095	1.8	1.91	Sugar ABC transporter ATP-binding protein
	SM_RS23980	4.16	2.17	Rhizopine uptake ABC transporter substrate-binding protein precursor
	SM_RS27100	1.79	2.08	NodJ ABC transporter permease
	SM_RS25295	1.74	1.76	ABC transporter substrate-binding protein
	SM_RS26280	1.8	1.9	Nitrate ABC transporter ATP-binding protein
	SM_RS11510	1.86	1.97	ABC transporter permease
	SM_RS13215	2.16	2.06	High-affinity branched-chain amino acid ABC transporter
	SM_RS26290	1.57	1.62	Nitrate ABC transporter substrate-binding protein
	SM_RS19540	2.09	2.04	Sugar ABC transporter permease
	SM_RS17120	1.7	1.87	Lactose ABC transporter permease
	SM_RS18870	1.55	1.72	Iron ABC transporter permease
	SM_RS19125	1.83	2.15	Glycerol-3-phosphate ABC transporter permease
Lipid metabolism	SM_RS21880	2.68	2.69	Hypothetical protein SM_b21670
	SM_RS29235	2.2	2.42	Hypothetical protein SMa1666
	SM_RS22185	2.15	1.85	GntR family transcriptional regulator
	SM_RS17520	2.15	2.32	Hypothetical protein SM_b20081
	SM_RS28255	1.93	1.9	VirB5 type IV secretion protein
	SM_RS29880	1.92	2.36	Oxidoreductase
	SM_RS22530	1.91	1.78	DedA family large C4-dicarboxylate uptake permease
	SM_RS20455	1.83	2.15	Hypothetical protein SM_b21090
	SM_RS13145	1.8	2.1	Transport transmembrane protein
	SM_RS13465	1.68	1.72	Transporter
	SM_RS30220	1.67	1.58	Hypothetical protein SMa2012
	SM_RS29760	1.63	1.68	Aldehyde
	SM_RS22030	1.62	1.95	Two-component sensor histidine kinase

FC1, Fold changes of BolA_CLas_/ΔBolA_Sme_ vs. ΔBolA_Sme_; FC2, Fold changes of Rm1021 vs. ΔBolA_Sme_.

**Figure 4:**
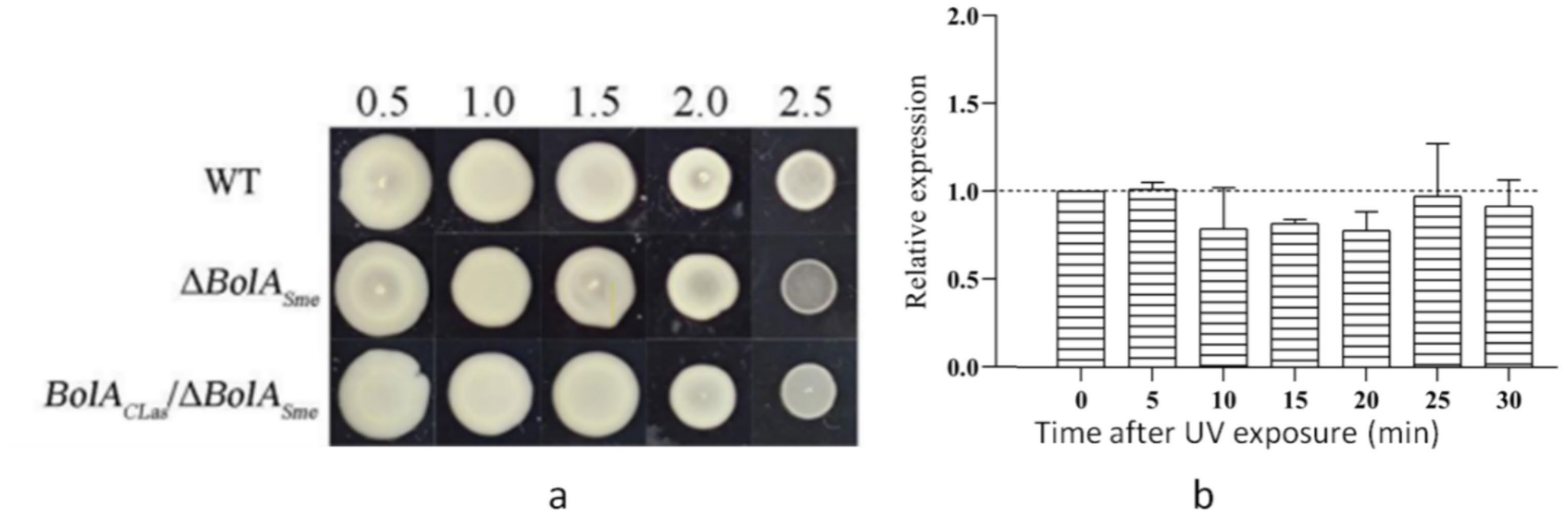
Effect of *BolA* deletion and complementation on stress tolerance. **(a)** Bacterial growth phenotypes under salt stress. **(b)** Relative expression level of the *BolA* in *Candidatus Liberibacter asiaticus* (CLas)-infected citrus leaves under ultraviolet (UV) stress treatment.

To assess the role of BolA in stress tolerance, salt and ultraviolet (UV) stress assays were performed. For salt tolerance, Sme strains were spotted on TY agar containing increasing concentrations of NaCl (0–2.5%). Colony size decreased gradually with rising salt concentration in all strains. On the TY medium with 2.5% NaCl, the Δ*BolA_Sme_* mutant exhibited markedly impaired growth compared to the wild-type strain, as judged by colony size. The *BolA*_CLas_/Δ*BolA_Sme_* complementation strain showed partial restoration of growth, with colony sizes intermediate between the mutant and wild-type (Figure 4a). While this qualitative observation supports a role for BolA in salt tolerance, future studies incorporating quantitative growth assays (e.g., CFU counts) will be necessary to fully corroborate this conclusion.

For the UV stress assay, the expression level of the *BolA* gene of CLas was quantified using RT-qPCR and normalized to the *gyrA*, which served as an internal reference. Contrary to the induction typically seen in some SOS-regulated genes in free-living bacteria, the expression of *BolA* was mildly downregulated in CLas-infected citrus leaves following transient UV exposure, showing a slight decrease at all time points examined (5, 10, 15, 20, 25, and 30 min post-exposure) (Figure 4b). This suggests that BolA may not be a component of the core SOS regulon in CLas.

### 3.5 BolA regulates the sec secretion system and serves as a new potential target for CLas pathogenicity

Transcriptomic analysis revealed that five heme metabolism-related DEGs (*hemA*, *hemB*, *hemF*, *hemH*, and *ubiG*) involved in cellular metabolic pathways were all upregulated in Δ*BolA_Sme_* (Table 3). Additionally, 14 DEGs associated with protein translocation, including key components of the Sec secretion system (*secA*, *ftsY*, *secD*/*secF*, *secB*, *secY*, *ffh*, and *yidC*), were also induced in Δ*BolA_Sme_*. These results suggest that BolA acts as a negative regulator of the Sec secretion pathway in both *Sme* and CLas. Furthermore, *BolA* appears to contribute to protein folding, sorting, and degradation, as indicated by the downregulation of genes such as *rho*, *dnaK*, *groEL*, and *str1* in both comparisons (*BolA*_CLas_/Δ*BolA_Sme_ vs*. Δ*BolA_Sme_* and Rm1021 *vs*. Δ*BolA_Sme_*).

**Table 3:** Differential expression genes of protein translocation and heme metabolic pathways were depressed by *BolA.*

Pathway	Gene ID	FC1	FC2	Description
Protein translocation	SM_RS13460	0.64	0.58	Preprotein translocase subunit SecA
	SM_RS17105	0.47	0.48	Transcription termination factor Rho
	SM_RS16600	0.54	0.51	Cell division protein
	SM_RS16615	0.51	0.59	Signal recognition particle protein
	SM_RS07340	0.28	0.37	Preprotein translocase subunit SecG
	SM_RS00940	0.28	0.42	Molecular chaperone DnaK
	SM_RS00035	0.57	0.58	Preprotein translocase subunit SecB
	SM_RS07020	0.49	0.4	Preprotein translocase subunit SecY
	SM_RS02260	0.46	0.47	Inner membrane protein translocase component YidC
	SM_RS26730	0.22	0.45	Molecular chaperone GroEL
	SM_RS04045	0.16	0.36	Chaperonin GroEL
	SM_RS08305	0.49	0.61	Thiosulfate sulfurtransferase
	SM_RS06525	0.59	0.44	Hypothetical protein SMc01929
	SM_RS02900	0.43	0.48	Preprotein translocase subunit SecD/SecF
Heme metabolic	SM_RS15315	0.66	0.58	5-aminolevulinate synthase
	SM_RS06120	0.36	0.34	Delta-aminolevulinic acid dehydratase
	SM_RS09390	0.4	0.46	Coproporphyrinogen III oxidase
	SM_RS14350	0.42	0.49	Ferrochelatase
	SM_RS13400	0.62	0.55	3-demethylubiquinone-9 3-methyltransferase

FC1, Fold changes of BolA_CLas_/ΔBolA_Sme_ vs. ΔBolA_Sme_; FC2, Fold changes of Rm1021 vs. ΔBolA_Sme_.

**Figure 5:**
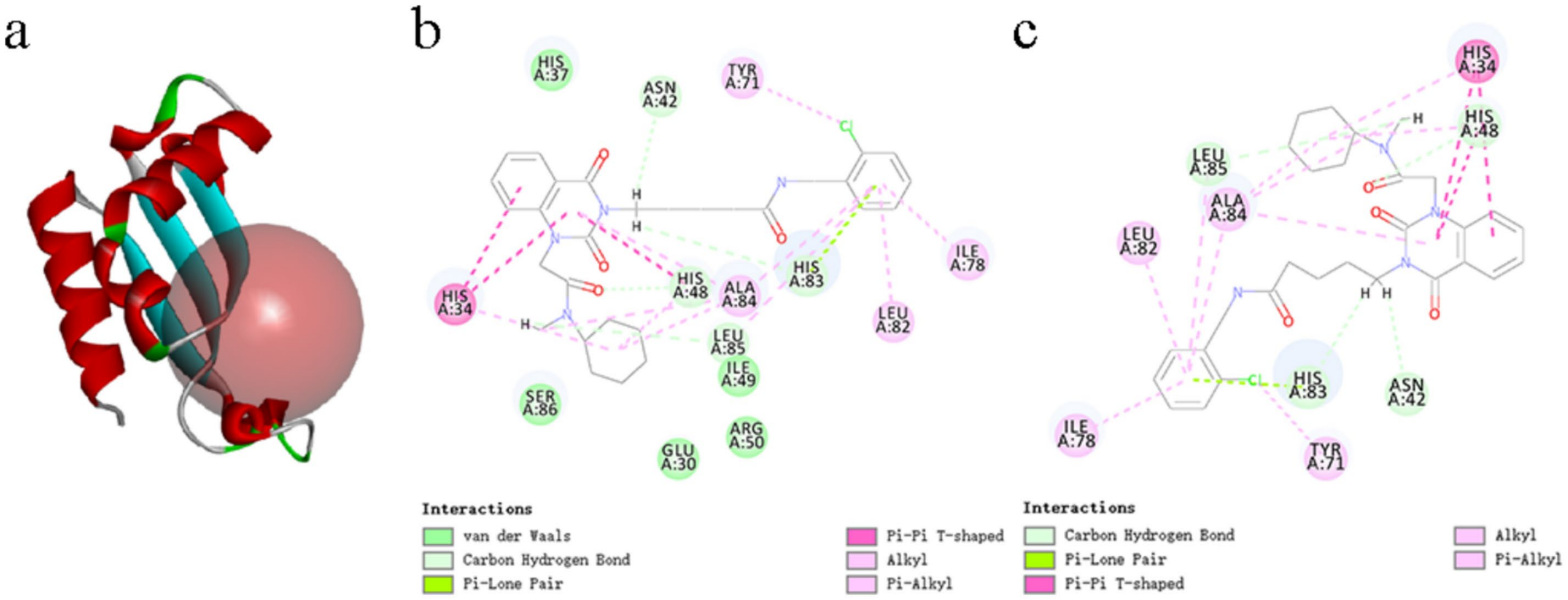
Homology model and molecular docking of CLas BolA: **(a)** Predicted three-dimensional structure of CLas BolA. The *α*-helices are shown in red, *β*-sheets in green, and the predicted active pocket is represented by a red sphere; **(b)** Molecular docking pose of compound B1 (N-[(2-chlorophenyl)methyl]-5-(1-{[cyclohexyl(methyl)carbamoyl]methyl}-2,4-dioxo-1,2,3,4-tetrahydroquinazolin-3-yl)pentanamide) within the active pocket of CLas BolA; **(c)** Molecular docking pose of compound B1 (2-({[(furan-2-yl)methyl]carbamoyl}methyl)sulfanyl]-4-oxo-N,3-bis[(oxolan-2-yl)methyl]-3,4-dihydroquinazoline-7-carboxamide) within the active pocket of CLas BolA.

To identify potential inhibitors of BolA, a three-dimensional homology model of the CLas BolA protein was generated (Figure 5a). Virtual screening of small molecule inhibitors targeting BolA in the HIT database was conducted. Two compounds with the highest scores, N-[(2-chlorophenyl)methyl]-5-(1-【[cyclohexyl(methyl) carbamoyl] methyl】-2,4-dioxo-1,2,3,4- tetrahydroquinazolin −3-yl) pentanamide and 2-[(【[(furan-2-yl) methyl] carbamoyl】methyl) sulfanyl]-4-oxo-N,3-bis[(oxolan-2-yl) methyl]-3,4-dihydroquinazoline-7-carboxamide (hereafter referred to as B1 and B2), were selected for molecular docking (Figures 5b,c). Given the uncultivable nature of CLas, we first evaluated the bioactivity of the candidate inhibitors by determining their MIC against *Sme*. Both compounds exhibited an MIC of 32.5 μg/mL against *Sme*, suggesting their ability to penetrate bacterial cells and inhibit growth in a preliminary screen.

**Figure 6:**
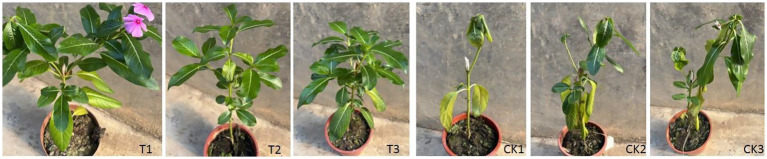
Phenotypic evaluation of periwinkle plants following graft-inoculation with CLas-infected leaves treatment with and without candidate BolA inhibitor B2.

The *in planta* efficacy of these compounds was evaluated using a grafting assay. CLas-infected periwinkle branches were cultured hydroponically for 20 days in solutions containing compound B2. Leaves from these branches were then grafted onto healthy periwinkle seedlings. A significant reduction in CLas titer was observed in the plants grafted with compound-treated leaves, with a Ct value of 30.32 ± 0.97 compared to the control plants grafted with leaves from branches cultivated in sterilized water (Ct value of 21.45 ± 1.06). The significant reduction in CLas titer following treatment demonstrates that pharmacological targeting of BolA can effectively control CLas growth within its host environment. The phenotypic outcome of the inhibitor treatment was visually corroborated at 45 days post-grafting. Representative photographs of periwinkle plants are presented in Figure 6. Control plants (CK1-3), grafted with infected tissue but cultivated in sterile water, exhibited pronounced yellowing, mottling, and wilting consistent with advanced HLB symptoms. In contrast, plants treated with BolA-targeting compounds (T1-3) maintained significantly healthier overall appearances, characterized by larger canopy size, predominantly green foliage, and only minor leaf yellowing.

CK1-3: 45-day plants after grafting with CLas-infected periwinkle leaf tissue cultivated in sterile water. T1-3: Plants grafted with CLas-infected tissue and cultivated in hydroponic solutions containing candidate BolA inhibitors (compounds specified in Section 3.5).

Taken together, these results establish BolA as a promising target for both studying CLas pathogenicity and developing control strategies. Our findings delineate a functional link between BolA, the Sec secretion system, and CLas virulence: (1) BolA transcriptionally represses the Sec secretion system, a key virulence apparatus; (2) Inhibition of BolA function is predicted to depress Sec-mediated effector secretion, disrupting bacterial fitness or host interaction; and (3) This disruption ultimately leads to reduced bacterial titers *in planta*, validating BolA as a promising therapeutic target.

## 4 Discussion

ABC transporters play an important role in the stress response (Nagayama et al., 2014) and regulate membrane transport under stress response (Herrou et al., 2017). No upregulation of zinc ABC transporters was observed in Δ*BolA_Sme_*. However, BolA can regulate lipid and iron ion ABC transporters, as well as some nutrient ABC transporters such as amino acids and sugars. In *Stenotrophomonas maltophilia*, iron plays a crucial role in regulating biofilm formation, oxidative stress response, expression of outer membrane protein, and bacterial virulence (Garcia et al., 2015). In the process of infection, pathogens need to regulate the metabolic pathway of iron to enhance their own virulence. Pathogens have evolved with various strategies to coordinate iron metabolism and virulence in response to changes in environmental iron to maintain iron homeostasis (Pandey et al., 2016). Since iron is largely sequestered in host tissue and predominantly binds to heme, pathogens take up the host’s heme, internalize the heme, and subsequently lyse it via oxidative and non-oxidative mechanisms to release iron (Huang and Wilks, 2017; Lyles and Eichenbaum, 2018). Zheng et al. (2023) found that the heme metabolic pathway was upregulated in PGD strains (lower *BolA* expression). We further suggest that the upregulation of heme metabolism is negatively regulated by BolA, which may be related to CLas survival and colonization or even virulence. Furthermore, the role of BolA in stress adaptation appears to extend to genotoxic stress. Contrary to the induction typical of some SOS-responsive genes, UV exposure led to a consistent downregulation of CLas *BolA* (Figure 4). This suggests that BolA may not be part of the core SOS regulon in this pathogen. Instead, the downregulation could represent a distinct resource-reallocation strategy, whereby CLas suppresses certain non-essential functions to prioritize energy for immediate survival and DNA repair under stress, underscoring its specialized adaptation as an obligate intracellular bacterium.

Lipids are a major class of biomolecules essential for cell maintenance and homeostasis. The movement of lipids within and between bacterial cell membranes is essential for building and maintaining bacterial cell envelopes (Giacometti et al., 2022). The DedA family is a conserved membrane protein family found in most organisms, and its members are essential for antibiotic resistance in *Burkholderia* (Panta et al., 2019). In this study, the expression of DedA was positively related to BolA and suggested to be involved in regulating the antibiotic resistance of CLas. The GntR family is one of the most common families of TFs, and its members are predicted to regulate bacterial virulence, the hypersensitive response (HR), and T3SS in *Xanthomonas citri* (Zhou et al., 2017) and in *Xanthomonas campestris* (Su et al., 2016). In this study, BolA induced the expression of a *GntR* gene, which also regulated the flagella motility and biofilm formation (Wassinger et al., 2013). The possible direct or indirect links of these two TFs and their role in the CLas require further verification.

Flagellar motility enables bacteria to escape unfavorable conditions and plays a key role in host colonization, biofilm formation, and other host interactions (Kolter and Greenberg, 2006). Although the presence of a flagellum has not been observed for CLas in citrus and dodder, a few CLas cells showed thread-like structures in psyllid guts, and 30 flagellate-related genes (3 clusters) have been predicted in the CLas genome. In addition, Las expresses flagellin protein in psyllids, but not *in planta* (Andrade et al., 2020). The BolA represses the expression of several flagellar proteins, most of which are components of the basal body of flagellar machinery (Jones and Aizawa, 1991). These results further suggest that BolA may affect the motility of *Sme* and CLas. This result also provides a new theoretical support for the possibility of flagellar expression under certain circumstances.

Our functional characterization of CLas BolA—repressing motility, promoting biofilm, and enhancing stress tolerance—aligns with its well-established role as a conserved lifestyle switch in bacteria (Dressaire et al., 2015; da Silva et al., 2023). For instance, recent studies in *Salmonella enterica* demonstrate that BolA coordinately regulates these exact processes to influence virulence and environmental adaptation (Chen et al., 2024). This functional conservation validates our use of the *Sme* heterologous system and underscores the relevance of BolA as a regulatory node in CLas.

The Sec-dependent secretory pathway assists in the secretion of most secretory proteins to the extracellular level and plays a critical role in the various cycles of bacterial growth (Segers and Anne, 2011). Core components such as secG, secY, secB, secA, and secYEG, channel proteins are important in the Sec secretion machinery (Sardis and Economou, 2010; Sibbald et al., 2010; Sala et al., 2014; Tsirigotaki et al., 2017; Neef et al., 2020; Zhang et al., 2024). Sec-dependent effectors are closely related to CLas virulence and play roles in counteracting the host immune responses (Yan et al., 2013; Clark et al., 2018; Zhang et al., 2020; Li et al., 2024). Our study reveals that BolA represses the Sec secretion pathway. Consequently, different from the role of BolA acting as a virulence factor in animals (Zhang et al., 2022), this transcriptional regulator in CLas was found to play an opposite function in CLas. Interestingly, the MIC values of our selected compounds are consistent with those of small-molecule inhibitors designed with SecA as a target. The bacteriostatic effect of small-molecule inhibitors designed to target *SecA* on *Sme* was consistent with that of streptomycin (Hu et al., 2016). Zuo et al. (2024) demonstrated that a Sec-dependent effector of CLas can be blocked by small molecules. However, further validation of the efficacy of small molecule inhibitors in the treatment of HLB in controlled environments is required before potential field application.

**Figure 7:**
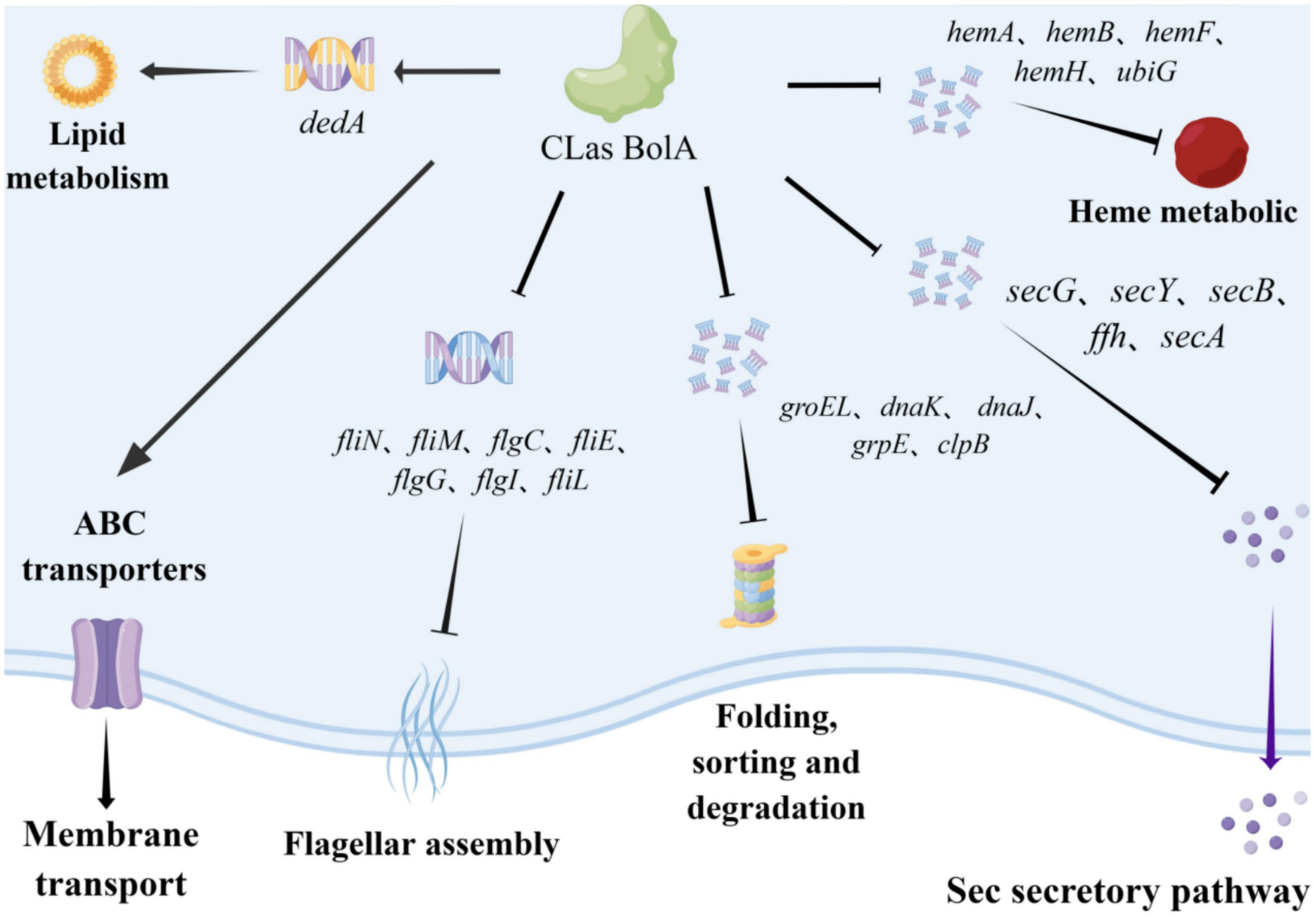
Predicted molecular regulatory mode of BolA-induced genes in “*Candidatus* Liberibacter asiaticus”.

Bacteria maintain protein homeostasis through molecular chaperones (e.g., *groEL*, *dnaK*, *dnaJ*, and *grpE*) and a molecular motor (Celaya et al., 2016). Among them, *clpB* is crucial for bacterial survival under diverse stress conditions (Alam et al., 2021). In this study, the protein depolymerization pathway was activated in the Δ*BolA_Sme_*, with *clpB* expression significantly upregulated. This upregulation may represent a compensatory response to restore intracellular homeostasis following disruption of the Sec secretion pathway upon *BolA* deletion, as illustrated in our proposed regulatory model (Figure 7).

Ultimately, targeting virulence regulators like BolA represents a promising pathogen-centric strategy within integrated HLB management frameworks (Ghosh et al., 2022). Our discovery that BolA inhibitors reduce CLas titers *in planta* provides a direct proof-of-concept. Future study should explore how BolA-mediated regulation, particularly of the Sec secretion system (Huang et al., 2024), interfaces with the host immune landscape (Ma et al., 2022) to refine such targeted interventions.

## 5 Conclusion

The transcriptional regulator BolA, which is differentially expressed in CLas strains of varying virulence, plays a central role in modulating multiple physiological and pathogenic traits. In this study, heterologous functional analysis in Sme revealed that BolA positively regulates biofilm formation and stress tolerance, while negatively controlling bacterial motility through repression of flagellar assembly. Additionally, BolA activates nutrient ABC transporters and lipid metabolism, but inhibits heme metabolism. Critically, we establish a direct functional link between BolA and the Sec secretion system—a key virulence apparatus in CLas. BolA transcriptionally represses the Sec system, and its pharmacological inhibition is predicted to disrupt Sec-mediated effector secretion, ultimately reducing bacterial titers in planta. This mechanistic insight, combined with the successful identification of BolA-targeting small-molecule inhibitors, validates BolA as a promising and druggable target for controlling citrus HLB. Consequently, we believe that BolA is a key transcription regulatory factor involved in virulence and stress response in CLas. The results of this study provide new ideas for the development of drugs targeting CLas genes.

## Data Availability

The datasets presented in this study can be found in online repositories. The names of the repository/repositories and accession number(s) can be found in the article/supplementary material.

## Supplementary material

SUPPLEMENTARY FIGURE 1 PCR verification of the BolASme gene deletion mutant and the recombinant suicide plasmid. **(a)** PCR identification of the wild-type Sme Rm1021 and the ΔBolASme mutant. Lane 1: wild-type Sme Rm1021; Lane 2: ΔBolASme mutant. **(b)** Construction and verification of the recombinant suicide plasmid pK18mobsacB/BolASme. Lane 3: PCR verification of the recombinant suicide plasmid pK18mobsacB/BolASme (showing a specific band); Lane 5: PCR negative control of the empty vector pK18mobsacB (no specific band); M: DNA size marker. (Lane 4 shows the result for another unrelated construct, which is not discussed herein).

## References

[ref1] Alam A. Broms J. E. Kumar R. Sjostedt A. (2021). The role of ClpB in bacterial stress responses and virulence. Front. Mol. Biosci. 8:668910. doi: 10.3389/fmolb.2021.668910, 33968993 PMC8100447

[ref2] Aldea M. Garrido T. Hernandez-Chico C. Vicente M. Kushner S. R. (1989). Induction of a growth-phase-dependent promoter triggers transcription of BolA, an *Escherichia coli* morphogene. EMBO J. 8, 3923–3931. doi: 10.1002/j.1460-2075.1989.tb08573.x, 2684651 PMC402084

[ref3] Andrade M. O. Pang Z. Achor D. S. Wang H. Yao T. Singer B. H. . (2020). The flagella of ‘*Candidatus* Liberibacter asiaticus’ and its movement in planta. Mol. Plant Pathol. 21, 109–123. doi: 10.1111/mpp.12884, 31721403 PMC6913195

[ref1001] Bao M. Zheng Z. Sun X. Chen J. Deng X. (2020). Enhancing PCR capacity to detect ‘Candidatus Liberibacter asiaticus’ utilizing whole genome sequence information. Plant Dis. 104, 527–532. doi: 10.1094/PDIS-05-19-0931-RE, 31790641

[ref4] Barnett M. J. Solow-Cordero D. E. Long S. R. (2019). A high-throughput system to identify inhibitors of *Candidatus* Liberibacter asiaticus transcription regulators. Proc. Natl. Acad. Sci. USA 116, 18009–18014. doi: 10.1073/pnas.1905149116, 31427509 PMC6731658

[ref5] Capela D. Barloy-Hubler F. Gouzy J. Bothe G. Ampe F. Batut J. . (2001). Analysis of the chromosome sequence of the legume symbiont *Sinorhizobium meliloti* strain 1021. Proc. Natl. Acad. Sci. U. S. A. 98, 9877–9882. doi: 10.1073/pnas.161294398, 11481430 PMC55546

[ref6] Celaya G. Fernandez-Higuero J. A. Martin I. Rivas G. Moro F. Muga A. (2016). Crowding modulates the conformation, affinity, and activity of the components of the bacterial disaggregate machinery. J. Mol. Biol. 428, 2474–2487. doi: 10.1016/j.jmb.2016.04.027, 27133933

[ref7] Chen Z. Huo X. Wan J. Che J. Deng M. Bao Y. . (2024). Enhancing acid resistance of *Escherichia coli* based on directed morphology evolutionary of key transcription factor bolA. Food Biosci. 62:105291. doi: 10.1016/j.fbio.2024.105291

[ref8] Chen K. Zhan Z. Li L. Li J. Zhou Z. Wang N. . (2024). BolA affects the biofilm formation ability, outer membrane permeability and virulence, thus is required for the adaptability of *Salmonella enterica* serotype Typhimurium to the harsh survival environment. Microbiol. Res. 274:127423. doi: 10.1016/j.micres.2023.127423, 37295142

[ref9] Clark K. Franco J. Y. Schwizer S. Pang Z. Hawara E. Liebrand T. W. H. . (2018). An effector from the huanglongbing-associated pathogen targets citrus proteases. Nat. Commun. 9:1718. doi: 10.1038/s41467-018-04140-9, 29712915 PMC5928222

[ref10] Da Silva A. A. Galego L. Arraiano C. M. (2023). New perspectives on BolA: a still mysterious protein connecting morphogenesis, biofilm production, virulence, Iron metabolism, and stress survival. Microorganisms 11:632. doi: 10.3390/microorganisms11030632, 36985206 PMC10051749

[ref11] Dressaire C. Moreira R. N. Barahona S. De Alves Matos A. P. Arraiano C. M. (2015). BolA is a transcriptional switch that turns off motility and turns on biofilm development. mBio 6:e02352-14. doi: 10.1128/mBio.02352-14, 25691594 PMC4337573

[ref12] Duan Y. Zhou L. Hall D. G. Li W. Doddapaneni H. Lin H. . (2009). Complete genome sequence of citrus huanglongbing bacterium, '*Candidatus* Liberibacter asiaticus' obtained through metagenomics. Mol. Plant-Microbe Interact. 22, 1011–1020. doi: 10.1094/MPMI-22-8-1011, 19589076

[ref14] Galibert F. Finan T. M. Long S. R. Puhler A. Abola P. Ampe F. . (2001). The composite genome of the legume symbiont *Sinorhizobium meliloti*. Science 293, 668–672. doi: 10.1126/science.1060966, 11474104

[ref15] Garcia C. A. Alcaraz E. S. Franco M. A. Passerini de Rossi B. N. (2015). Iron is a signal for *Stenotrophomonas maltophilia* biofilm formation, oxidative stress response, OMPs expression, and virulence. Front. Microbiol. 6:926. doi: 10.3389/fmicb.2015.00926, 26388863 PMC4559654

[ref16] Garnier M. Jagoueix-Eveillard S. Cronje P. R. Le Roux H. F. Bove J. M. (2000). Genomic characterization of a liberibacter present in an ornamental rutaceous tree, *Calodendrum capense*, in the Western Cape Province of South Africa. Proposal of ‘*Candidatus* Liberibacter africanus subsp. *capensis*’. Int. J. Syst. Evol. Microbiol. 50 Pt 6, 2119–2125. doi: 10.1099/00207713-50-6-2119, 11155987

[ref17] Ghosh D. Kokane S. Savita B. K. Kumar P. Sharma A. K. Ozcan A. . (2022). Huanglongbing pandemic: current challenges and emerging management strategies. Plants 12:160. doi: 10.3390/plants12010160, 36616289 PMC9824665

[ref18] Giacometti S. I. MacRae M. R. Dancel-Manning K. Bhabha G. Ekiert D. C. (2022). Lipid transport across bacterial membranes. Annu. Rev. Cell Dev. Biol. 38, 125–153. doi: 10.1146/annurev-cellbio-120420-022914, 35850151 PMC12981311

[ref19] Herrou J. Willett J. W. Czyz D. M. Babnigg G. Kim Y. Crosson S. (2017). Conserved ABC transport system regulated by the general stress response pathways of alpha- and Gammaproteobacteria. J. Bacteriol. 199:e00746-16. doi: 10.1128/JB.00746-16, 27994018 PMC5309909

[ref20] Hu J. Akula N. Wang N. (2016). Development of a microemulsion formulation for antimicrobial SecA inhibitors. PLoS One 11:e0150433. doi: 10.1371/journal.pone.0150433, 26963811 PMC4786163

[ref21] Huang G. Chang X. Hu Y. Li F. Wang N. Li R. (2024). SDE19, a sec-dependent effector from ‘*Candidatus* Liberibacter asiaticus’ suppresses plant immunity and targets *Citrus sinensis* Sec12 to interfere with vesicle trafficking. PLoS Pathog. 20:e1012542. doi: 10.1371/journal.ppat.1012542, 39255299 PMC11414923

[ref22] Huang W. Wilks A. (2017). Extracellular heme uptake and the challenge of bacterial cell membranes. Annu. Rev. Biochem. 86, 799–823. doi: 10.1146/annurev-biochem-060815-014214, 28426241

[ref23] Jones C. J. Aizawa S. (1991). The bacterial flagellum and flagellar motor: structure, assembly and function. Adv. Microb. Physiol. 32, 109–172. doi: 10.1016/S0065-2911(08)60007-7, 1882727

[ref24] Kolter R. Greenberg E. P. (2006). Microbial sciences: the superficial life of microbes. Nature 441, 300–302. doi: 10.1038/441300a, 16710410

[ref25] Li X. Guo Z. Zhou Y. Zhang B. Ruan H. Chen W. (2024). Three new discovery effector proteins from *Candidatus* Liberibacter asiaticus psy62 inhibit plant defense through interaction with AtCAT3 and AtGAPA. Plant Cell Rep. 43:130. doi: 10.1007/s00299-024-03220-z, 38652336

[ref26] Lyles K. V. Eichenbaum Z. (2018). From host heme to iron: the expanding spectrum of heme degrading enzymes used by pathogenic bacteria. Front. Cell. Infect. Microbiol. 8:198. doi: 10.3389/fcimb.2018.00198, 29971218 PMC6018153

[ref27] Ma W. Pang Z. Huang X. Xu J. Pandey S. S. Li J. . (2022). Citrus huanglongbing is a pathogen-triggered immune disease that can be mitigated with antioxidants and gibberellin. Nat. Commun. 13:529. doi: 10.1038/s41467-022-28189-9, 35082290 PMC8791970

[ref28] Mil-Homens D. Barahona S. Moreira R. N. Silva I. J. Pinto S. N. Fialho A. M. . (2018). Stress response protein BolA influences fitness and promotes *Salmonella enterica* serovar Typhimurium virulence. Appl. Environ. Microbiol. 84, e02850–e02817. doi: 10.1128/AEM.02850-17, 29439986 PMC5881071

[ref29] Nagayama K. Fujita K. Takashima Y. Ardin A. C. Ooshima T. Matsumoto-Nakano M. (2014). Role of ABC transporter proteins in stress responses of Streptococcus mutants. Oral Health Dent. Manag. 13, 359–365, 24984648

[ref30] Neef J. Bongiorni C. Schmidt B. Goosens V. J. van Dijl J. M. (2020). Relative contributions of non-essential sec pathway components and cell envelope-associated proteases to high-level enzyme secretion by *Bacillus subtilis*. Microb. Cell Factories 19:52. doi: 10.1186/s12934-020-01315-2, 32111210 PMC7048088

[ref31] Nehela Y. Killiny N. (2020). Revisiting the complex pathosystem of huanglongbing: deciphering the role of citrus metabolites in symptom development. Meta 10:409. doi: 10.3390/metabo10100409, 33066072 PMC7600524

[ref32] Occhialini A. Cunnac S. Reymond N. Genin S. Boucher C. (2005). Genome-wide analysis of gene expression in *Ralstonia solanacearum* reveals that the hrpB gene acts as a regulatory switch controlling multiple virulence pathways. Mol. Plant Microbe Interact. 18, 938–949. doi: 10.1094/MPMI-18-0938, 16167764

[ref33] Pagliai F. A. Pan L. Silva D. Gonzalez C. F. Lorca G. L. (2018). Zinc is an inhibitor of the LdtR transcriptional activator. PLoS One 13:e0195746. doi: 10.1371/journal.pone.0195746, 29634775 PMC5892913

[ref34] Pandey S. S. Patnana P. K. Lomada S. K. Tomar A. Chatterjee S. (2016). Co-regulation of iron metabolism and virulence associated functions by iron and XibR, a novel iron binding transcription factor, in the plant pathogen *Xanthomonas*. PLoS Pathog. 12:e1006019. doi: 10.1371/journal.ppat.1006019, 27902780 PMC5130282

[ref35] Panta P. R. Kumar S. Stafford C. F. Billiot C. E. Douglass M. V. Herrera C. M. . (2019). A DedA family membrane protein is required for *Burkholderia thailandensis* colistin resistance. Front. Microbiol. 10:2532. doi: 10.3389/fmicb.2019.02532, 31827463 PMC6849406

[ref36] Sala A. Bordes P. Genevaux P. (2014). Multitasking SecB chaperones in bacteria. Front. Microbiol. 5:666. doi: 10.3389/fmicb.2014.00666, 25538690 PMC4257090

[ref37] Sardis M. F. Economou A. (2010). SecA: a tale of two protomers. Mol. Microbiol. 76, 1070–1081. doi: 10.1111/j.1365-2958.2010.07176.x, 20444093

[ref38] Schafer A. Tauch A. Jager W. Kalinowski J. Thierbach G. Puhler A. (1994). Small mobilizable multi-purpose cloning vectors derived from the *Escherichia coli* plasmids pK18 and pK19: selection of defined deletions in the chromosome of *Corynebacterium glutamicum*. Gene 145, 69–73. doi: 10.1016/0378-1119(94)90324-7 8045426

[ref39] Segers K. Anne J. (2011). Traffic jam at the bacterial sec translocase: targeting the SecA nanomotor by small-molecule inhibitors. Chem. Biol. 18, 685–698. doi: 10.1016/j.chembiol.2011.04.007, 21700205

[ref40] Sibbald M. J. Winter T. van der Kooi-Pol M. M. Buist G. Tsompanidou E. Bosma T. . (2010). Synthetic effects of secG and secY2 mutations on exoproteome biogenesis in *Staphylococcus aureus*. J. Bacteriol. 192, 3788–3800. doi: 10.1128/JB.01452-09 20472795 PMC2897339

[ref41] Su H. Z. Wu L. Qi Y. H. Liu G. F. Lu G. T. Tang J. L. (2016). Characterization of the GntR family regulator HpaR1 of the crucifer black rot pathogen *Xanthomonas campestris* pathovar campestris. Sci. Rep. 6:19862. doi: 10.1038/srep19862, 26818230 PMC4730234

[ref42] Talib E. A. Outten C. E. (2021). Iron-sulfur cluster biogenesis, trafficking, and signaling: roles for CGFS glutaredoxins and BolA proteins. Biochim. Biophys. Acta, Mol. Cell Res. 1868:118847. doi: 10.1016/j.bbamcr.2020.118847, 32910989 PMC7837452

[ref43] Tsirigotaki A. De Geyter J. Sostaric N. Economou A. Karamanou S. (2017). Protein export through the bacterial sec pathway. Nat. Rev. Microbiol. 15, 21–36. doi: 10.1038/nrmicro.2016.161, 27890920

[ref44] Vahling-Armstrong C. M. Zhou H. Benyon L. Morgan J. K. Duan Y. (2012). Two plant bacteria, S. Meliloti and ca. Liberibacter asiaticus, share functional znuABC homologues that encode for a high affinity zinc uptake system. PLoS One 7:e37340. doi: 10.1371/journal.pone.0037340, 22655039 PMC3360030

[ref45] Wang N. Trivedi P. (2013). Citrus huanglongbing: a newly relevant disease presents unprecedented challenges. Phytopathology 103, 652–665. doi: 10.1094/PHYTO-12-12-0331-RVW, 23441969

[ref46] Wang W. Xu J. Wang N. (2025). Functional characterization of transcriptional regulator rem in ‘*Candidatus* Liberibacter asiaticus’. Phytopathology 115, 454–468. doi: 10.1094/PHYTO-10-24-0339-R, 39891894

[ref47] Wassinger A. Zhang L. Tracy E. Munson R. S. Kathariou S. Wang H. H. (2013). Role of a GntR-family response regulator LbrA in *Listeria monocytogenes* biofilm formation. PLoS One 8:e70448. doi: 10.1371/journal.pone.0070448, 23894658 PMC3720924

[ref48] Waterhouse A. Bertoni M. Bienert S. Studer G. Tauriello G. Gumienny R. . (2018). SWISS-MODEL: homology modelling of protein structures and complexes. Nucleic Acids Res. 46, W296–W303. doi: 10.1093/nar/gky427, 29788355 PMC6030848

[ref1002] Xu M. X. Chen S. K. Guan T. B Ren F. M. Teng J. F. Tan X. Q. Discovery of anti‑Pseudomonas aeruginosa components from traditional Chinese medicine based on virtual screening. Chemical Research and Application, (2022) 34, 754–763., 35082290

[ref49] Yan Q. Sreedharan A. Wei S. Wang J. Pelz-Stelinski K. Folimonova S. . (2013). Global gene expression changes in *Candidatus* Liberibacter asiaticus during the transmission in distinct hosts between plant and insect. Mol. Plant Pathol. 14, 391–404. doi: 10.1111/mpp.12015, 23336388 PMC6638839

[ref50] Zhang C. Du P. Yan H. Zhu Z. Wang X. Li W. (2020). A sec-dependent secretory protein of the huanglongbing-associated pathogen suppresses hypersensitive cell death in *Nicotiana benthamiana*. Front. Microbiol. 11:594669. doi: 10.3389/fmicb.2020.594669, 33329478 PMC7734103

[ref51] Zhang S. Wang X. Zhao T. Zhou C. (2024). Effector C Las0185 targets methionine sulphoxide reductase B1 of *Citrus sinensis* to promote multiplication of ‘*Candidatus* Liberibacter asiaticus’ via enhancing enzymatic activity of ascorbate peroxidase 1. Mol. Plant Pathol. 25:e70002. doi: 10.1111/mpp.70002, 39215961 PMC11365454

[ref52] Zhang F. Yan X. Bai J. Xiang L. Ding M. Li Q. . (2022). Identification of the BolA protein reveals a novel virulence factor in *K. pneumoniae* that contributes to survival in host. Microbiol. Spectr. 10:e0037822. doi: 10.1128/spectrum.00378-22, 36121239 PMC9603091

[ref53] Zheng Y. Zhang J. Li Y. Liu Y. Liang J. Wang C. . (2023). Pathogenicity and transcriptomic analyses of two "*Candidatus* Liberibacter asiaticus" strains harboring different types of phages. Microbiol. Spectr. 11:e0075423. doi: 10.1128/spectrum.00754-23 37071011 PMC10269750

[ref54] Zhou X. Yan Q. Wang N. (2017). Deciphering the regulon of a GntR family regulator via transcriptome and ChIP-exo analyses and its contribution to virulence in *Xanthomonas citri*. Mol. Plant Pathol. 18, 249–262. doi: 10.1111/mpp.12397, 26972728 PMC6638223

[ref55] Zou H. Gowda S. Zhou L. Hajeri S. Chen G. Duan Y. (2012). The destructive citrus pathogen, '*Candidatus* Liberibacter asiaticus' encodes a functional flagellin characteristic of a pathogen-associated molecular pattern. PLoS One 7:e46447. doi: 10.1371/journal.pone.0046447, 23029520 PMC3460909

[ref56] Zuo S. Xu L. Zhang H. Jiang M. Wu S. Zhang L. H. . (2024). FlgI is a sec-dependent effector of *Candidatus* Liberibacter asiaticus that can be blocked by small molecules identified using a yeast screen. Plants 13:318. doi: 10.3390/plants13020318, 38276775 PMC10819201

