# Correction: *Candidatus* Liberibacter asiaticus encodes a functional BolA transcriptional regulator related to motility, biofilm development, and stress response

**DOI:** 10.3389/fmicb.2026.1820587

**Published:** 2026-04-07

**Authors:** Xuanlin Zhan, Guoyi Huang, Jun Su, Jingtian Zhang, Qiting Huang, Xiaoling Deng, Meirong Xu

**Affiliations:** 1 Guangdong Province Key Laboratory of Microbial Signals and Disease Control, Citrus Huanglongbing Research Laboratory, South China Agricultural University, Guangzhou, China; 2 National Center for International Collaboration Research on Precision Agricultural Aviation Pesticide Spraying Technology, Guangdong Engineering Technology Research Center of Smart Agriculture, College of Artificial Intelligence, South China Agricultural University, Guangzhou, China

**Keywords:** citrus greening, gene function, heterologous expression, huanglongbing, inhibitor screening, virulence

Affiliation “National Center for International Collaboration Research on Precision Agricultural Aviation Pesticide Spraying Technology, Guangdong Engineering Technology Research Center of Smart Agriculture, College of Artificial Intelligence, South China Agricultural University, Guangzhou, China” was omitted for author Xiaoling Deng.

This affiliation has now been added for author Xiaoling Deng.

Author Meirong Xu was erroneously assigned as sole corresponding author.

The correct corresponding authors are Xiaoling Deng and Meirong Xu.

The funder “National Natural Science Foundation of China, Grant No. 32371984” to author Xiaoling Deng was erroneously omitted.

The correct **Funding** statement is below:

“The author(s) declared that financial support was received for this work and/or its publication. This research was supported by Modern Agriculture Technology Systems and Chinese Modern Agricultural Technology Systems (Grant/Award No. CARS-26) and by National Natural Science Foundation of China, Grant No. 32371984 to Xiaoling Deng.”

The original version of this article has been updated.

