# Supplementary figures and images for: Candidatus Liberibacter asiaticus encodes a functional BolA transcriptional regulator related to motility, biofilm development, and stress response
Source: Front Microbiol. 2026 Jan 29;17:1717228. doi: 10.3389/fmicb.2026.1717228 (PMC12894273; doi:10.3389/fmicb.2026.1717228)

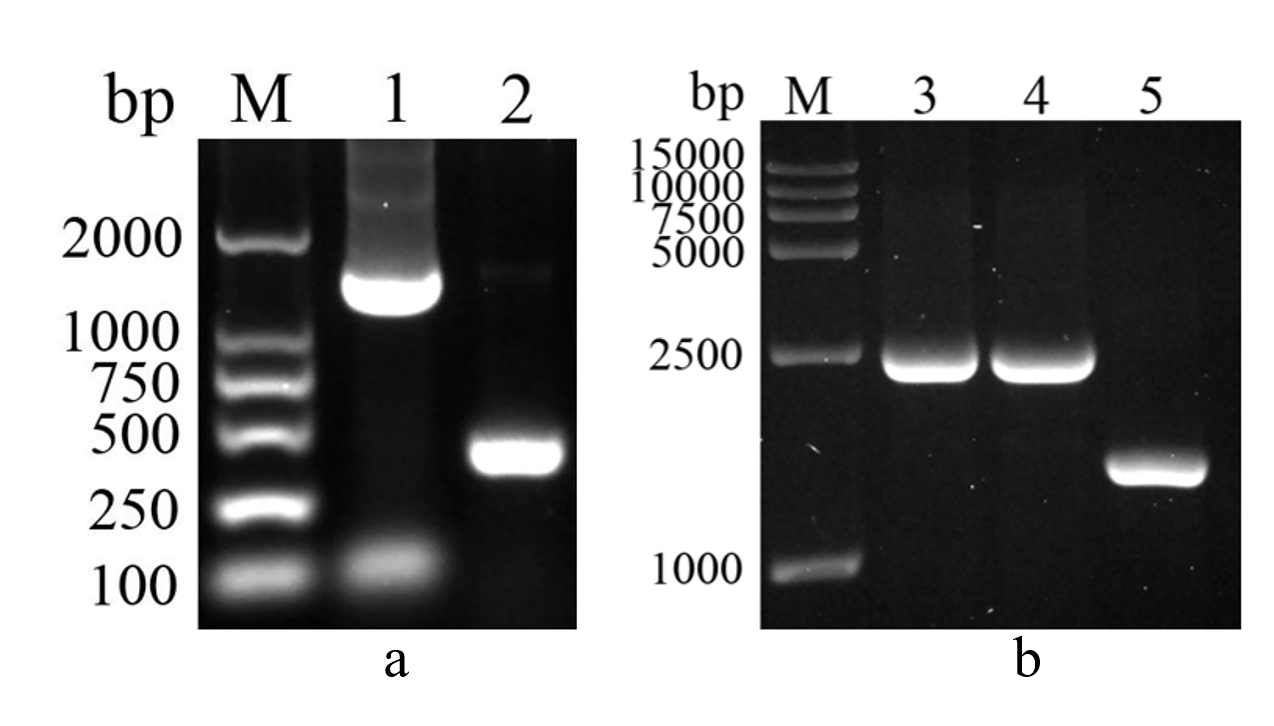

Supplement: SUPPLEMENTARY FIGURE 1 — PCR verification of the BolASme gene deletion mutant and the recombinant suicide plasmid. (a) PCR identification of the wild-type Sme Rm1021 and the ΔBolASme mutant. Lane 1: wild-type Sme Rm1021; Lane 2: ΔBolASme mutant. (b) Construction and verification of the recombinant suicide plasmid pK18mobsacB/BolASme. Lane 3: PCR verification of the recombinant suicide plasmid pK18mobsacB/BolASme (showing a specific band); Lane 5: PCR negative control of the empty vector pK18mobsacB (no specific band); M: DNA size marker. (Lane 4 shows the result for another unrelated construct, which is not discussed herein). [file Image_1.png]
